# Supplementary material for: Inhibitory Effects of Selected Medicinal Plants on Bacterial Growth of Methicillin-Resistant Staphylococcus aureus
Source: Molecules. 2022 Nov 11;27(22):7780. doi: 10.3390/molecules27227780 (PMC9693998; doi:10.3390/molecules27227780)
Supplement: Supplementary file 1 [file molecules-27-07780-s001.zip › molecules-2034392-Supplementary.pdf]

Supplementary Materials

Inhibitory effects of selected medicinal plants on bacterial growth of methicillin-resistant *Staphylococcus aureus*

In Geun Jung, Jae Young Jeong, Seung Hoon Yum, You Jin Hwang

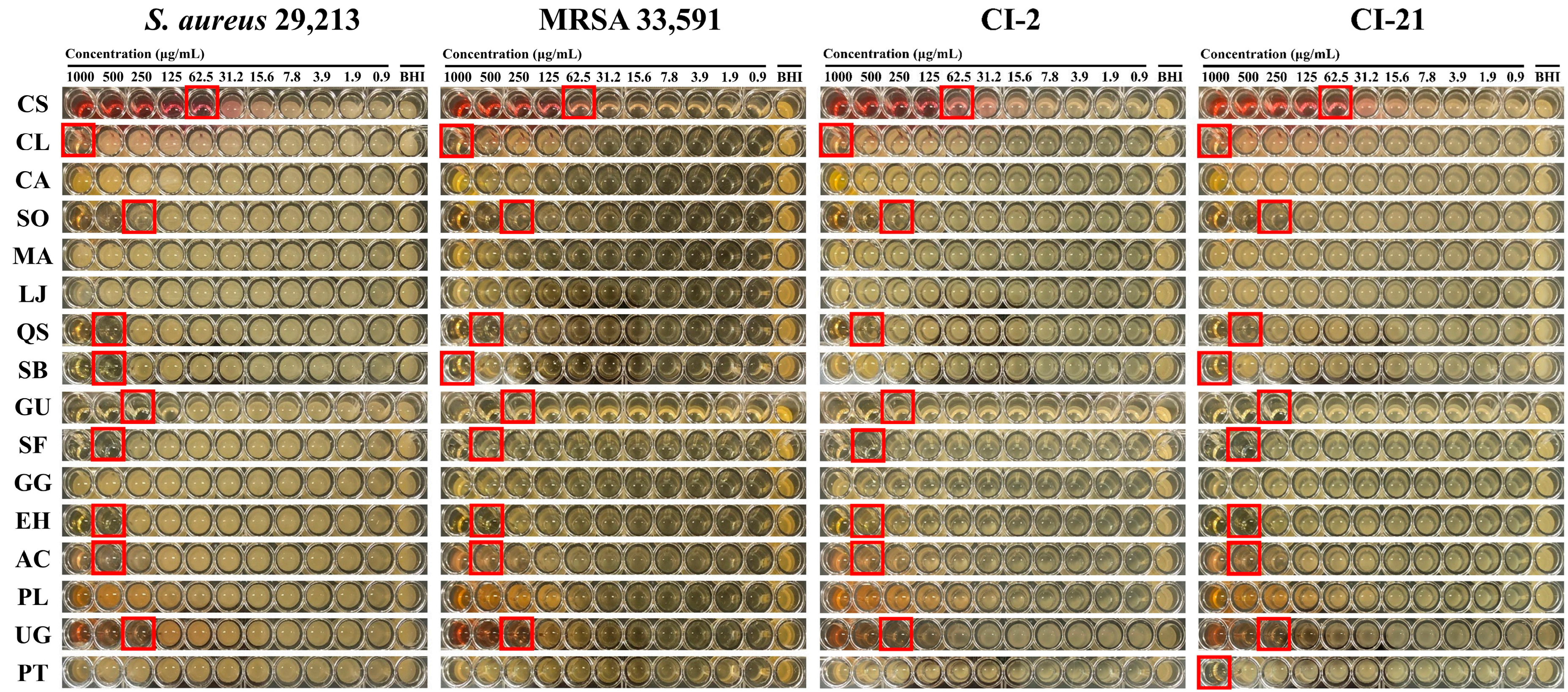

Supplementary Figure S1. Screening of ethanol extracts from 16 medicinal plants for antibacterial activities by broth microdilution method. Red squares are presented as MIC. *S. aureus*: *Staphylococcus aureus*; MRSA: Methicillin-resistant *Staphylococcus aureus*; CI: Clinical isolate; CS: *Caesalpinia sappan* L.; CL: *Cinnamomum loureirii* Nees.; CA: *Curcuma aromatica* Salisb.; SO: *Sanguisorba officinalis* L.; MA: *Morus alba* L.; LJ: *Lonicera japonica* Thunb; QS: *Quercus salicina* Blume; SB: *Scutellaria baicalensis* Georgi.; GU: *Glycyrrhiza uralensis* Fisch.; SF: *Sophora flavescens* Ait.; GG: *Glechoma grandis* (A. Gray) Kuprian.; EH: *Euphorbia humifusa* Wild.; AC: *Areca catechu* L.; PL: *Phellinus linteus*; UG: *Uncaria gambir* Roxb; PT: *Polygonum tinctorium* Ait.

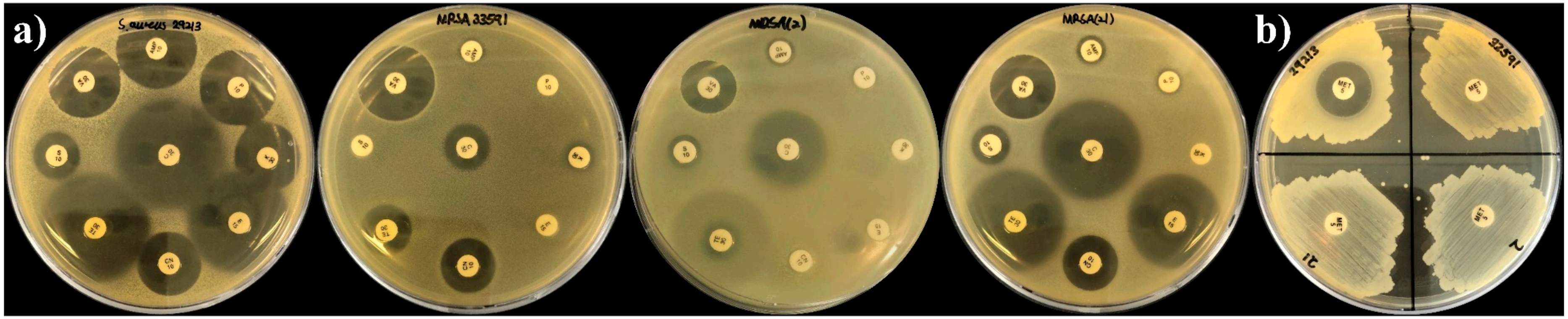

Supplementary Figure S2. Antibiotic susceptibility testing for *S. aureus* strains. a) Susceptibility for 9 different antibiotic discs; b) Susceptibility for methicillin antibiotic discs.

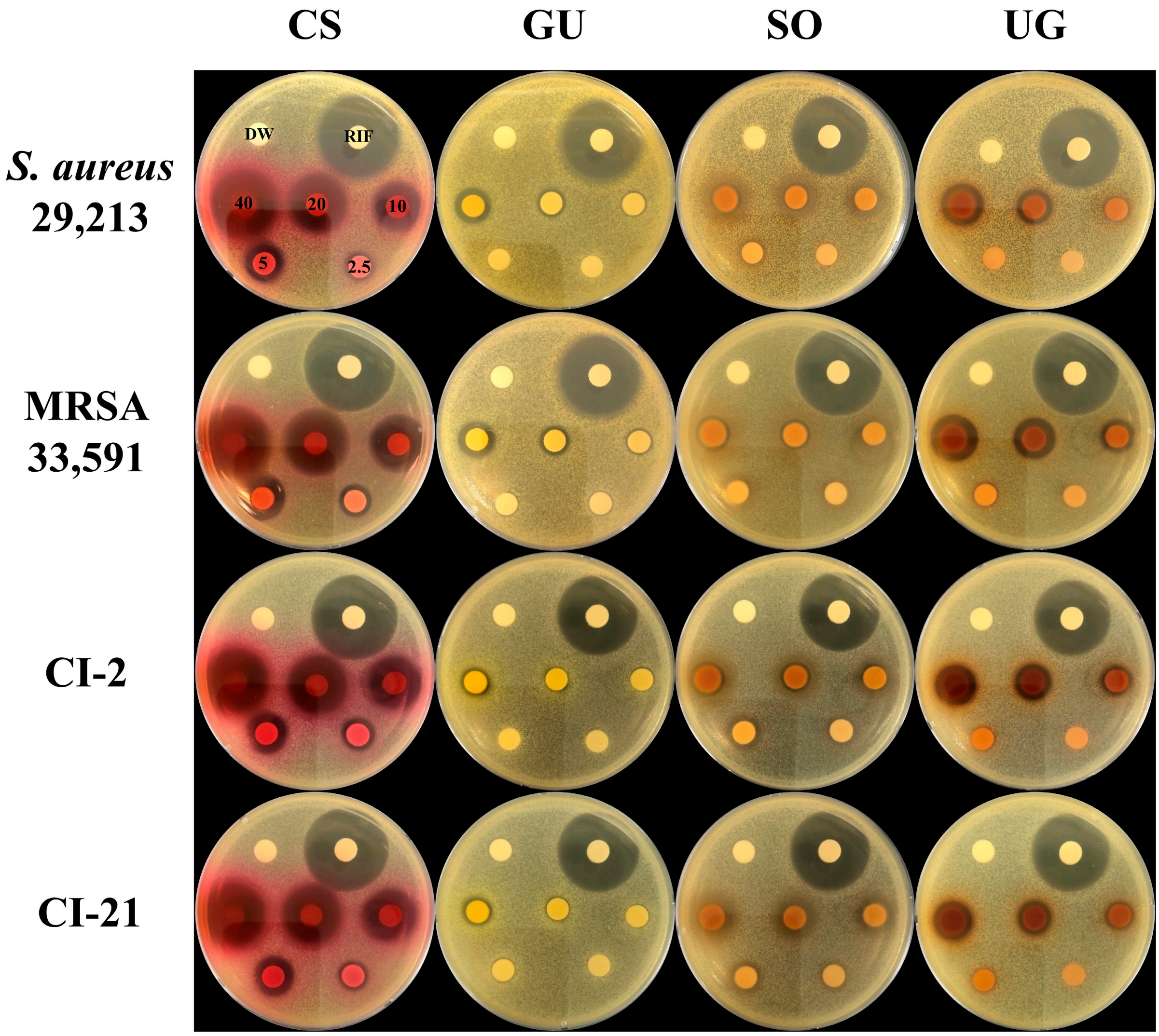

Supplementary Figure S3. Antibacterial activities of ethanol extracts from selected medicinal plants by disc diffusion assay. *S. aureus*: *Staphylococcus aureus*; MRSA: Methicillin-resistant *Staphylococcus aureus*; CI: Clinical isolate; CS: *Caesalpinia sappan* L.; GU: *Glycyrrhiza uralensis* Fisch.; SO: *Sanguisorba officinalis* L.; UG: *Uncaria gambir* Roxb. Briefly, each bacterial suspension (1 x 10^8 CFU/mL) was mixed well with 100 mL of fresh BHI agar medium. Then, 100 µL of each extract was loaded onto each paper-disc (8 mm/diameter). The concentration of ethanol extracts was 40, 20, 10, 5, and 2.5 mg/mL. Distilled water (DW) was served as a negative control, while rifampicin (RIF; 30 µL) was a positive control.

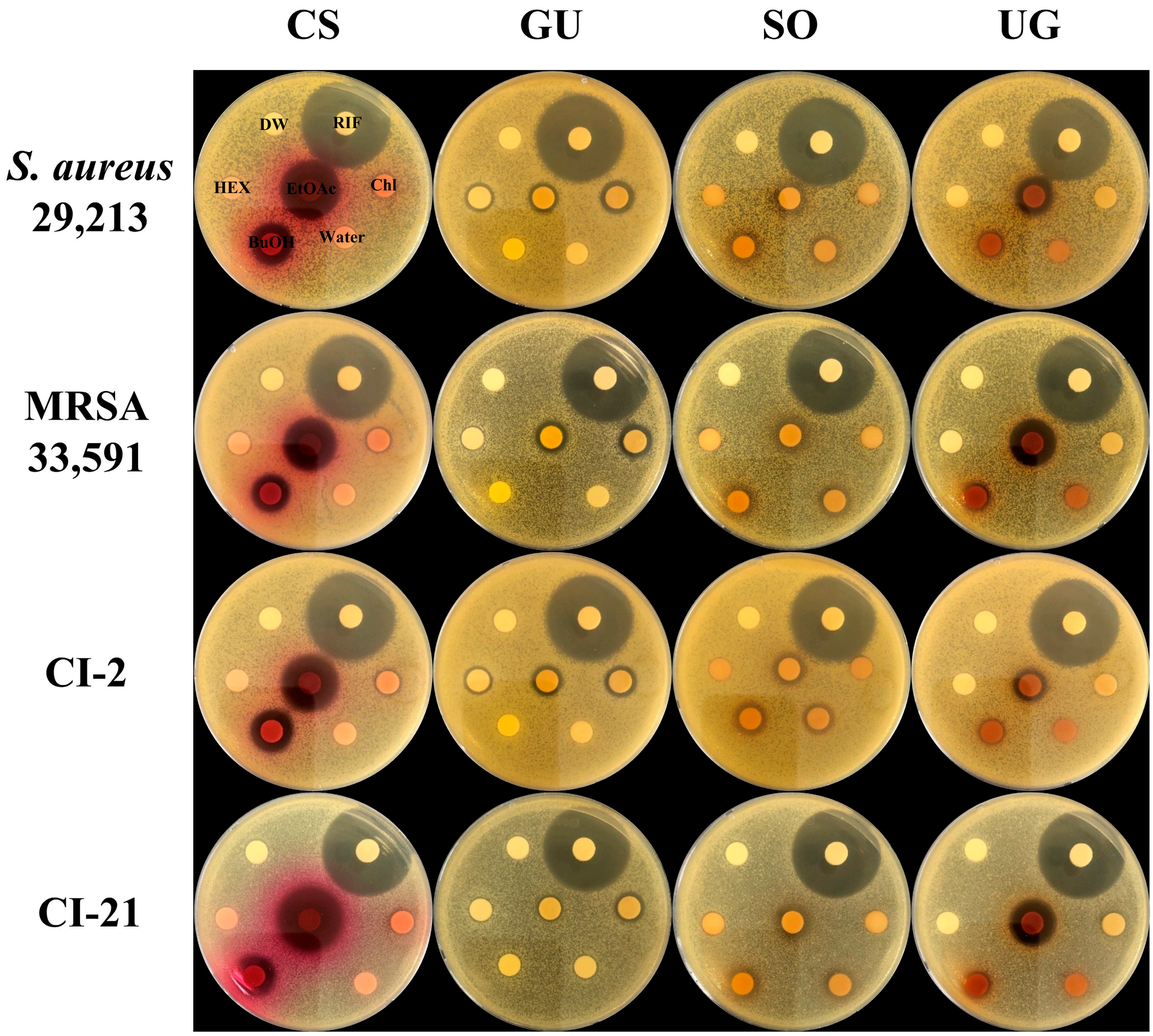

Supplementary Figure S4. Antibacterial activities of fractions from selected medicinal plants by disc diffusion assay. *S. aureus*: *Staphylococcus aureus*; MRSA: Methicillin-resistant *Staphylococcus aureus*; CI: Clinical isolate; CS: *Caesalpinia sappan* L.; GU: *Glycyrrhiza uralensis* Fisch.; SO: *Sanguisorba officinalis* L.; UG: *Uncaria gambir* Roxb. HEX: n-hexane; Chl: Chloroform; EtOAc: Ethyl acetate; BuOH: 1-butanol; Water: Distilled water. Briefly, each bacterial suspension (1 x 10^8 CFU/mL) was mixed well with 100 mL of fresh BHI agar medium. Then, 100 μL of each fraction was loaded onto paper-discs (8 mm/diameter). The concentration of fractions was 10 mg/mL. Distilled water (DW) was served as a negative-control, while rifampicin (RIF; 30 μL) was a positive-control.
